# Determinants of testosterone levels in human male obesity

**DOI:** 10.1007/s12020-015-0563-4

**Published:** 2015-03-13

**Authors:** Marlies Bekaert, Yves Van Nieuwenhove, Patrick Calders, Claude A. Cuvelier, Arsène-Hélène Batens, Jean-Marc Kaufman, D. Margriet Ouwens, Johannes B. Ruige

**Affiliations:** 1 Department of Endocrinology, Ghent University Hospital, De Pintelaan 185, Building 6 K12, 9000 Ghent, Belgium; 2 Department of Gastrointestinal Surgery, Ghent University Hospital, 9000 Ghent, Belgium; 3 Revalidation Science and Physiotherapy, Ghent University Hospital, 9000 Ghent, Belgium; 4 Department of Pathology, Ghent University Hospital, 9000 Ghent, Belgium; 5 Institute for Clinical Biochemistry and Pathobiochemistry, German Diabetes Center, 40225 Dūsseldorf, Germany

**Keywords:** Aromatase, Adipocyte cell size, Obesity, Type 2 diabetes

## Abstract

Testosterone (T) levels are decreased in obese men, but the underlying causes are incompletely understood. Our objective was to explore the relation between low (free) T levels and male obesity, by evaluating metabolic parameters, subcutaneous adipose tissue (SAT) aromatase expression, and parameters of the hypothalamic–pituitary–gonadal axis. We recruited 57 morbidly obese men [33 had type 2 diabetes (DM2)] and 25 normal-weight men undergoing abdominal surgery. Fourteen obese men also attended a follow-up, 2 years after gastric bypass surgery (GBS). Circulating T levels were quantified by LC–MS/MS, whereas free T levels were measured using serum equilibrium dialysis and sex hormone-binding globulin, luteinizing hormone, and follicle-stimulating hormone by immunoassay. SAT biopsies were used to determine adipocyte cell size and aromatase expression by real-time PCR. Total and free T levels were decreased in obese males versus controls, with a further decrease in obese men with DM2 versus obese men without DM2. There were no differences in aromatase expression among the study groups, and sex steroids did not correlate with aromatase expression. Pearson analysis revealed an inverse association between (free) T and SAT cell size, triglycerides, and HOMA-IR. Multivariate analysis confirmed the inverse association between (free) T and SAT cell size (*β* = −0.321, *P* = 0.037 and *β* = −0.441, *P* = 0.011, respectively), independent of age, triglycerides, HOMA-IR, obesity, or diabetes. T levels were normalized 2 years after GBS. These data suggest that SAT cell size rather than SAT aromatase expression or parameters of the hypothalamic–pituitary–gonadal axis is related to low T in male obesity, which points to adipose cell size-related metabolic changes as a major trigger in decreased T levels.

## Introduction

The relationship between decreased testosterone (T) levels and male obesity is incompletely understood. In general, the synthesis of T by Leydig cells in the testis is stimulated by luteinizing hormone (LH) together with follicle-stimulating hormone (FSH). Estradiol (E_2_) is known to be an important down-regulator of serum T levels by inhibiting the release of these gonadotropins from the pituitary gland [1, 2]. Some studies suggest that an increased activity of aromatase, the enzyme converting T into E_2_, in adipose tissue may contribute to elevated E_2_ levels in obese men [3, 4]. However, data on E_2_ levels in obese men are inconsistent, as some studies reported increased E_2_ levels, while others reported no changes or even lower levels [5–7]. Alternatively, abdominal obesity itself may contribute to the decline in circulating T levels [8, 9]. Obesity leads to an expansion of fat (hypertrophy of adipocytes), which is associated with a deteriorated metabolic profile, including glucose intolerance, dyslipidemia, hypertension, and inflammation [10, 11]. Prospective studies have shown that both adiposity and the presence of the metabolic syndrome are predictive of future low T levels and could accelerate the age-related decline of T [12, 13]. Consistently, weight loss has led to increased T levels in obese men [14, 15]. Although the underlying mechanisms are still unclear, these findings suggest that hypertrophy of the adipocytes and its related metabolic changes may associate with the decline in T levels in obese men.

This cross-sectional study aimed to explore potential determinants of (free) T levels in a cohort of morbidly obese men with and without type 2 diabetes as well as men with normal body weight. Specifically, associations with adipose tissue aromatase expression levels, subcutaneous adipose tissue (SAT) cell size, and insulin resistance and triglyceride (TG) levels (marker of adiposity) were examined. Furthermore, the effects of weight loss following gastric bypass surgery (GBS) on (free) T levels and potential determinants thereof were examined in morbidly obese men at a follow-up examination 2 years after GBS.

## Research design and methods

### Subjects

The study cohort consisted of twenty-five normal-weight and fifty-seven morbidly obese men. Thirty-three of the morbidly obese men had type 2 diabetes according to the American Diabetes Association (ADA) criteria [16]. All men were scheduled for abdominal surgery. The obese men underwent GBS, and normal-weight men had surgery for adhesiolysis, rupture of the stomach, intestinal resection, stomach closing, or Nissen fundoplication. Obesity was defined as BMI >30 kg/m^2^. Although having abdominal surgery, the normal-weight men had an overall good health. Participants with primary hypogonadism, abnormal thyroid function, hepatitis or malignancies, serum total cholesterol >300 mg/dl, and/or serum TG >450 mg/dl were excluded. None of the subjects used steroids, and oral glucose-lowering medication were discontinued prior to surgery. A subgroup of thirty-six patients that underwent GBS was invited for a follow-up examination, when 2 years had passed since surgery. Fourteen of them were willing to participate (39 %). Subjects attending follow-up were not using sex steroids or other androgen-related drugs, and three out of fourteen subjects were using metformin medication. The study was approved by the institutional ethics committee, and participants gave their written informed consent, which was validated by the Ethical Review Board of Ghent University Hospital and conducted according to the principles of the Declaration of Helsinki (Registration no: B67020084018).

At baseline and during follow-up, clinical and anthropometric parameters were assessed as described previously [17]. The fat percentage of body weight (fat %) was estimated by bio-impedance (Bodystat 1500, Bodystat, Ltd, Isle of Man, UK).

### Hormonal and biochemical assays

Blood samples were collected from the patients after overnight fasting, prior to surgery. Serum samples were centrifuged, fractionated, and stored at −80 °C until analysis. Fasting TG, glucose, and insulin levels were measured using standard laboratory assays (modular immunoassay, Roche Diagnostics, Mannheim, Germany). HOMA-IR was calculated with the following formula: (fasting glucose [mmol/L] × fasting insulin [µU/mL])/22.5] [18]. Total T and E_2_ were measured with liquid chromatography tandem mass spectrometry (LC–MS/MS). Serum T levels were analyzed using a Waters C-18 acquity ultra-performance liquid chromatography (UPLC) column (Waters Corporation, Milford, MA, USA), with a limit of quantification (LOQ) of 0.087 nmol/L (CV <20 %, *n* ≥ 6). Intra- and inter-assay coefficients of variation (CV) were 9.1 % at 0.48 nmol/L and 7.3 % at 1.35 nmol/L, respectively. For determination of E_2_ levels, 2D-LC–MS/MS was performed on an AB Sciex 5500 triple-quadrupole mass spectrometer (AB Sciex, Toronto, Canada) as described by Fiers et al. [19]. LOQ (CV <20 %, *n* ≥ 6) could be ascertained at 1.1 pmol/L for E_2_, and intra- and inter-assay CV were 3.7 % at 69.4 pmol/L and 4.0 % at 77.4 pmol/L, respectively. Free T levels were measured using a validated equilibrium dialysis method, as described previously by Vermeulen et al. [20]. Free E_2_ levels were calculated from total E_2_, sex hormone-binding globulin (SHBG), and albumin concentrations as described elsewhere [21]. Commercial immunoassays were used to determine SHBG (Orion Diagnostica, Espoo, Finland), LH, and FSH (Elecsys LH and FSH immunoassay; Roche Diagnostics).

### Adipose tissue processing

**Fig. 1:**
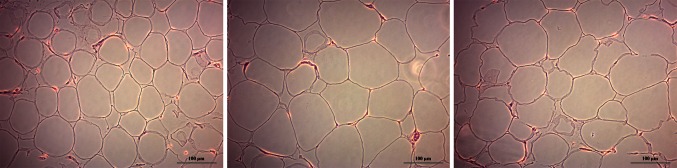
Digital photographs of the subcutaneous adipose tissue (SAT) paraffin slides from a control and obese subject without and with type 2 diabetes, respectively. Photographs were taken with an AxioCam ERc 5s camera placed on an Axioskop 20 light microscope at ×20 magnification. Mean surface area of adipocytes was measured using the ZEN 2011 software by indicating margins of all complete adipocytes imaged on the slides, expressed in µm^2^. Presented images were randomly selected. *Scale bar* represents 100 µm

SAT biopsies were obtained at the end of the surgical intervention and stored at −80 °C until further analysis, or fixated in formol (buffered 4 % paraformaldehyde solution; Klinipath, Belgium) at room temperature for microscopic analysis. Fixation, dehydration, cleaning, and paraffin impregnation (Tissue Tek Vip, Sakura, USA) of these samples were performed, followed by embedding with a TBS 88 Paraffin Embedding System (Medite, USA). By means of a Tissue Tek Prisma (Sakura), Hematoxylin–eosin staining and film coverslipping of 3 μm slides were completed. Digital photographs of the paraffin slides were taken with an AxioCam ERc 5s camera and Axioskop 20 light microscope (Zeiss, Jena, Germany) at ×20 magnification, with a total of 6 photographs per slide (Fig. 1). The surface area of by average 143 adipocytes per slide was then measured using the ZEN 2011 software (Zeiss) by indicating the margins of the cell membrane of all complete adipocytes. As adipocytes were assumed to be spheres, as many as possible complete imaged adipocytes were measured in order to calculate the median surface area as expressed in µm^2^ per study patient, followed by calculation of the median SAT cell size per study group. SAT cell size assessment was blinded to grouping and was determined in 50 subjects from the cross-sectional study cohort.

Aromatase expression was determined in the frozen SAT samples of 36 subjects using real-time polymerase chain reaction (real-time PCR). First, RNA was isolated out of 100 mg of the frozen fat biopsies with the Tripure Isolation Reagent kit (Roche Diagnostics) according to the manufacturer’s instructions. Contaminating genomic DNA was removed with the RNase-Free DNase Set (Qiagen, Hilden, Germany), followed by a clean-up step with the RNeasy Mini Kit (Qiagen). cDNA synthesis was carried out using the SuperScript First-Strand Synthesis System for RT-PCR kit (Invitrogen, Ghent, Belgium) according to the manufacturer’s instructions. Aromatase expression levels were quantified by real-time PCR using a Quantitect CYP19A1 primer assay (Qiagen) and SYBR Green PCR Master Mix (Applied Biosystems, Foster City, CA, USA) on a StepOne Plus system (Applied Biosystems). The obtained threshold cycle (*C*_t_) values were normalized for the expression of the stable reference gene RPS18 (Quantitect RPS18 primer assay; Qiagen) using the Δ*C*_t_-method.

### Statistical analysis

The statistical analysis was performed using IBM SPSS Statistics (version 20.0). Data distribution was evaluated with the Kolmogorov–Smirnov test. Variables displaying a normal distribution were expressed as mean ± SD, whereas non-Gaussian distributed variables were described as median (interquartile range). ANOVA and Tukey HSD post hoc test were used for the comparison of variables among non-obese men, morbidly obese men, and morbidly obese men with type 2 diabetes of the cross-sectional study. Non-Gaussian distributed variables were tested using Kruskal–Wallis and Mann–Whitney *U* test. Pearson correlation coefficients were calculated for the whole study samples as well as for the subgroups (normal-weight men and obese men with and without type 2 diabetes) and were adjusted for age. If not normally distributed, the variables underwent a logarithmic transformation prior to analysis. Multivariate linear regression analysis was used to identify independent factors associated with T levels in men, using a model containing significant variables from univariate analysis. Regression analysis was corrected for grouping by adding obesity and type 2 diabetes as independent binary variables (yes/no) to the model. Prior to regression analysis, variables were standardized in order to retrieve standard error (SE) and 95 % confidence intervals (95 % CI) on *β* coefficients. Effects after bariatric surgery were analyzed using a paired student *t* test or Wilcoxon matched-pairs signed-ranks test in case of non-parametric data distribution. *P* values <0.05 (two-tailed) were considered statistically significant.

## Results

### Subject characteristics

Characteristics of the study participants are listed in Table 1. Briefly, obese men without type 2 diabetes were younger as compared to the normal-weight men and obese men with type 2 diabetes (41 [32–49] versus 49 [43–64] versus 54 [50–61] years, respectively; (median[Q1–Q3]) *P* = 0.001). Apart from a higher BMI, the obese men were insulin resistant and had an increased fasting glucose, fasting insulin, and fasting TG levels versus normal-weight men. In obese men with type 2 diabetes, BMI, HOMA-IR, and fasting insulin as well as fasting glucose levels were even higher as compared to obese men without type 2 diabetes. Finally, although SAT cell size was similar between obese men with and without type 2 diabetes, it almost doubled versus normal-weight men (Table 1).

**Table 1:** Clinical and laboratory data of the study cohort, including control, obese, and obese subjects with DM2

Parameter	Controls (*N* = 25)	Obese (*N* = 24)	Obese + DM2 (*N* = 33)	*P*	*P*_control versus obese_	*P*_control versus obese + DM2_	*P*_obese versus obese + DM2_
*Clinical and biochemical characteristics*							
Age (years)	49 [43–64]	41 [32–49]	54 [50–61]	0.001	0.015	0.350	<0.001
BMI (kg/m^2^)	24 ± 4	41 ± 6	44 ± 6	<0.001	<0.001	<0.001	0.051
Glucose (mmol/L)	4.72 [4.08–5.30]	5.33 [4.77–5.61]	6.99 [6.30–9.10]	<0.001	0.014	<0.001	<0.001
Insulin (pmol/L)	35.9 [22.3–64.6]	114.8 [61.8–179.4]	197.9 [122.2–278.0]	<0.001	<0.001	<0.001	0.003
Triglycerides (mmol/L)	1.20 [0.81–2.27]	2.10 [1.30–3.18]	1.76 [1.34–2.66]	0.015	0.012	0.011	0.588
HOMA-IR	0.64 [0.40–1.15]	1.99 [1.07–3.19]	4.02 [2.56–4.90]	<0.001	<0.001	<0.001	0.001
SAT cell size^a^ (µm^2^)	3346 ± 1494	6370 ± 1009	5765 ± 1192	<0.001	<0.001	<0.001	0.381
*Sex steroids*							
SAT aromatase^b^ (AU)	94.0 [60.0–148.0]	137.0 [70.0–226.0]	92.5 [55.3–244.0]	0.546	0.237	0.702	0.547
Testosterone (nmol/L)	16.36 ± 6.74	10.81 ± 5.09	7.17 ± 2.90	<0.001	0.001	<0.001	0.024
Estradiol (pmol/L)	63.9 [45.1–88.6]	87.1 [57.8–103.1]	67.5 [52.5–96.0]	0.180	0.060	0.460	0.247
Free testosterone (pmol/L)	295.0 ± 138.7	239.5 ± 109.0	164.1 ± 69.9	<0.001	0.174	<0.001	0.029
Free estradiol (pmol/L)	1.17 [0.77–1.67]	1.61 [1.13–2.15]	1.32 [1.07–1.90]	0.076	0.024	0.165	0.310
SHBG (nmol/L)	41.0 [32.3–49.0]	22.4 [18.2–29.3]	24.0 [16.4–27.9]	<0.001	<0.001	<0.001	0.771
LH (IU/L)	5.0 [3.6–8.0]	4.0 [3.0–5.1]	4.6 [2.8–6.8]	0.347	0.143	0.714	0.298
FSH (IU/L)	7.2 [4.3–17.3]	4.2 [2.9–5.6]	5.1 [3.6–10.5]	0.024	0.010	0.173	0.076

BMI, SAT cell size, T, and Free T were analyzed by one-way anova and Tukey HSD post hoc test. Non-Gaussian distributed variables were tested using Kruskal–Wallis test and Mann–Whitney *U* test. SAT subcutaneous adipose tissue; *DM2* type 2 diabetes

^a^SAT cell size was determined in *N* = 16 controls, *N* = 12 obese men without DM2, and *N* = 22 obese men with DM2

^b^SAT aromatase expression was determined in *N* = 11 controls, *N* = 11 obese men without DM2, and *N* = 14 obese men with DM2

Obese men with type 2 diabetes had lowest T levels versus obese men without type 2 diabetes and normal-weight men, respectively (7.17, 10.81, and 16.36 nmol/L, respectively; *P* < 0.001). Similarly, free T levels were lowest in obese men with type 2 diabetes versus obese men without type 2 diabetes and normal-weight men, respectively (164.1, 239.5, and 295.0 pmol/L, respectively; *P* < 0.001). There were no differences in circulating E_2_ levels and SAT aromatase expression among the groups. Finally, levels of FSH and SHBG were 35 and 43 % lower in obese men, respectively, irrespective of type 2 diabetes, whereas LH levels were similar among the groups (Table 1).

### Associations with testosterone levels

**Fig. 2:**
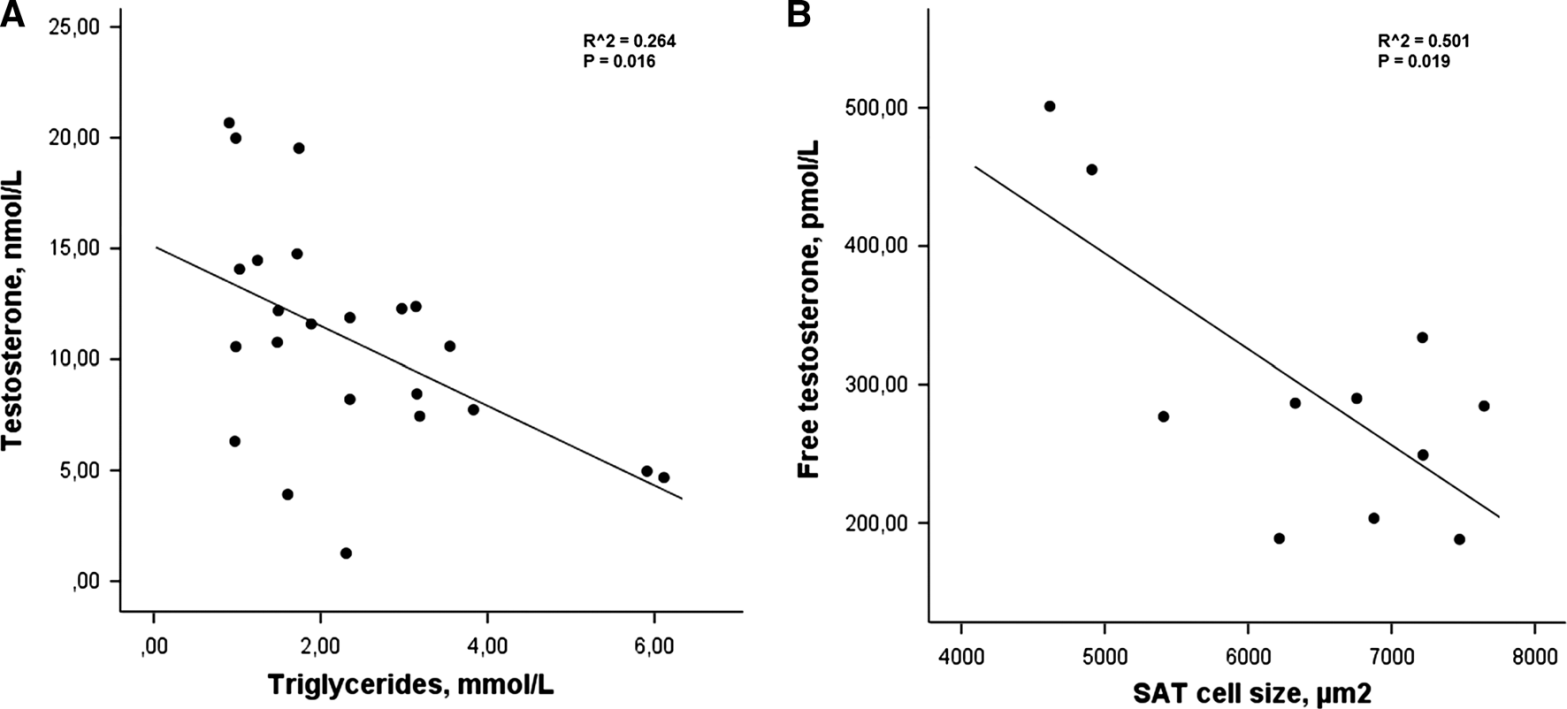
The relationship between triglycerides and serum testosterone levels (*n* = 23; **a**) as well as inverse associations between free testosterone and subcutaneous adipose tissue (SAT) cell size (*n* = 11; **b**) in obese men without type 2 diabetes

To identify potential mechanisms underlying the decrease in circulating (free) T levels in obese men, we performed a univariate correlation analysis. SAT aromatase expression showed no associations with sex steroids or with metabolic parameters neither in the whole study sample nor when analyzing subgroups (latter data not shown). In contrast, T was strongly negative associated with TG levels (*r* = −0.390, *P* < 0.001), HOMA-IR (*r* = −0.444, *P* < 0.001), and SAT cell size (*r* = −0.619, *P* < 0.001), in the whole study cohort. Similarly, free T levels were also negatively associated with TG levels (*r* = −0.242, *P* = 0.033), HOMA-IR (*r* = −0.286, *P* = 0.013), and SAT cell size (*r* = −0.599, *P* < 0.001) (Table 2). All correlation analyses were adjusted for age. An additional BMI adjustment retained the inverse association between T levels and TG levels (*r* = −0.235, *P* = 0.041), as well as between free T levels and SAT cell size (*r* = −0.340, *P* = 0.020). Furthermore, subgroup analyses revealed that the inverse correlation between total T levels and TG (*r* = −0.506, *P* = 0.016), as well as between free T levels and SAT cell size (*r* = −0.719, *P* = 0.019) (Fig. 2), remained significant in obese men without type 2 diabetes. Finally, when T-to-E_2_ ratio (ratio T/E_2_) was considered, similar results were found. Apart from the obvious relations to E_2_, ratio T/E_2_ was negatively associated with TG (*r* = −0.388, *P* < 0.001), HOMA-IR (*r* = −0.606, *P* < 0.001), and SAT cell size (*r* = −0.648, *P* < 0.001). Furthermore, there was a negative association with SAT aromatase, though this association was less strong (*r* = −0.343; *P* = 0.047) (Table 2).

**Table 2:** Associations between sex steroids, aromatase, and metabolic parameters

Parameter	Testosterone	*P* value	Free testosterone	*P* value	Ratio T/E_2_	*P* value	*N*
SAT aromatase	−0.095	0.600	0.027	0.879	−**0.343**	**0.047**	36
LH	0.187	0.104	0.061	0.597	0.112	0.326	82
FSH	0.216	0.064	0.088	0.452	0.173	0.136	82
SHBG	**0.694**	**<0.001**	**0.383**	**0.001**	**0.604**	**<0.001**	82
E_2_	0.211	0.064	0.214	0.056	−**0.308**	**0.005**	82
Free E_2_	0.054	0.637	0.162	0.151	−**0.463**	**<0.001**	82
TG	−**0.390**	**<0.001**	−**0.242**	**0.033**	−**0.388**	**<0.001**	82
HOMA−IR	−**0.444**	**<0.001**	−**0.286**	**0.013**	−**0.606**	**<0.001**	82
SAT cell size	−**0.619**	**<0.001**	−**0.599**	**<0.001**	−**0.648**	**<0.001**	50

Significant *P* values were indicated in bold

Data are Pearson correlation coefficients (*r*) adjusted for age. In case of non-Gaussian distribution, variables were log-transformed. *LH* luteinizing hormone, *E*_*2*_ estradiol, *TG* triglycerides, *HOMA-IR* homeostasis model of assessment for insulin resistance, *SAT* subcutaneous adipose tissue

To further substantiate the above findings, multivariate linear regression analysis was applied. Parameters showing statistical significance in univariate analysis were entered as variables of interest, after controlling for age and grouping (control versus obesity/diabetes). Regression analysis confirmed associations between T levels and SAT cell size (*β* = −0.324, *P* = 0.040) in a model which contained age, grouping, TG levels, and HOMA-IR. An identical model with free T levels and T/E_2_ ratio (the latter model contained also SAT aromatase considering its statistical significance in univariate analysis) as dependent variable showed similar results (*β* = −0.446, *P* = 0.013 and *β* = −0.391, *P* = 0.051, respectively) (Table 3).

**Table 3:** Multivariate linear regression model of variables significantly associated with total and free testosterone levels as well as testosterone-to-estradiol ratio (ratio T/E_2_) as dependent variables

Variable	*β*	SE (*β*)	95 % CI	*P* value
*Total testosterone*				
Obesity	−0.153	0.194	−0.546; 0.240	0.435
DM2	−0.617	0.220	−1.061; −0.173	**0.008**
Age	−0.171	0.118	−0.410; 0.068	0.157
Triglycerides	−0.054	0.106	−0.269; 0.160	0.610
HOMA-IR	−0.015	0.168	−0.354; 0.324	0.929
SAT cell size	−0.324	0.153	−0.633; −0.015	**0.040**
*Free testosterone*				
Obesity	0.049	0.218	−0.391; 0.489	0.822
DM2	−0.239	0.246	−0.735; 0.258	0.337
Age	−0.472	0.132	−0.740; −0.205	**0.001**
Triglycerides	0.043	0.119	−0.197; 0.282	0.722
HOMA-IR	−0.077	0.188	−0.457; 0.302	0.683
SAT cell size	−0.446	0.171	−0.791; −0.100	**0.013**
*Ratio* *T*/*E*_2_				
Obesity	−0.183	0.274	−0.772; 0.357	0.455
DM2	−0.519	0.323	−1.271; 0.061	0.073
Age	0.089	0.211	−0.325; 0.543	0.610
Triglycerides	−0.158	0.143	−0.437; 0.153	0.330
HOMA-IR	0.116	0.302	−0.493; 0.750	0.673
SAT cell size	−0.391	0.218	−0.897; 0.002	**0.051**
SAT aromatase	−0.167	0.184	−0.565; 0.192	0.319

Significant *P* values were indicated in bold

All *β* coefficients were standardized. *N* = 50

Obesity and DM2 are binary variables (yes/no). *DM2* type 2 diabetes, *HOMA-IR* homeostasis model of assessment for insulin resistance, *SAT* subcutaneous adipose tissue, *SE* standard error, *CI* confidence interval

### Characteristics and sex steroid levels of study participants after GBS

Fourteen men agreed to undergo a follow-up investigation 2 years after GBS. Table 4 displays their characteristics and sex steroid levels at baseline and 2 years after GBS. Metabolic characteristics of subjects who participated in the prospective study changed with a significant improvement of BMI (45–34 kg/m^2^), fat mass (45–36 %), TG (borderline; 1.55–1.09 mmol/L), glucose (6.47–5.35 mmol/L) and insulin (122.0–61.1 pmol/L) concentrations, and insulin sensitivity (HOMA-IR 3.3–1.1). Mean total T levels increased from 8.99 to 14.62 nmol/L (*P* = 0.004), SHBG levels increased from 27.9 to 52.4 nmol/L (*P* < 0.001), and FSH levels increased from 5.7 to 7.4 IU/L (*P* < 0.001). No significant differences could be established for free T or E_2_ before and after bariatric surgery. There were no associations of ∆ (free) T levels with any of the improved parameters (latter data not shown).

**Table 4:** Subject characteristics and sex steroid levels before and after bariatric surgery

	Pre-bariatric surgery (*N* = 14)	Post-bariatric surgery (*N* = 14)	*P* value
*Clinical and biochemical characteristics*			
Age (years)	51 ± 12	53 ± 12	<0.001
BMI (kg/m^2^)	45 ± 8	34 ± 8	<0.001
Fat (%)	45 ± 9	36 ± 12	0.009
Triglycerides (mmol/L)	1.55 ± 0.79	1.09 ± 0.46	0.067
Glucose (mmol/L)	6.47 ± 1.69	5.35 ± 0.74	0.051
Insulin (pmol/L)	122.0 [99.7–211.7]	61.1 [49.9–121.3]	0.001
HOMA-IR	3.3 [2.1–4.2]	1.1 [0.9–2.3]	0.002
*Sex steroids*			
Testosterone (nmol/L)	8.99 ± 4.70	14.62 ± 6.65	0.004
Free testosterone (pmol/L)	189.6 ± 110.5	229.0 ± 116.8	0.157
Estradiol (pmol/L)	70.4 ± 32.1	68.8 ± 25.9	0.848
Free estradiol (pmol/L)	1.33 ± 0.52	1.11 ± 0.44	0.117
SHBG (nmol/L)	27.9 [17.0–36.0]	52.4 [39.6–59.1]	<0.001
LH (IU/L)	5.2 [3.0–9.3]	5.8 [2.7–12.6]	0.832
FSH (IU/L)	5.7 [5.0–12.0]	7.4 [5.7–18.4]	<0.001

Data are mean ± SD or median (1^st^–3^rd^ quartile) in case of non-Gaussian distribution. *P* values were determined using a paired student *t* test. Non-Gaussian distributed variables were log-transformed

## Discussion

The present study showed a link between enlarged SAT cell size and low T levels in male obesity and could not establish a predominant role for adipose tissue aromatase expression and parameters of the hypothalamic–pituitary–gonadal (HPG) axis. SAT cell size was independently inversely associated with both total and free T levels after multivariate regression analysis corrected for age, grouping (control versus obesity/diabetes), HOMA-IR, and TG levels. Overall, the findings suggest that low T in male obesity might be related to enlargement of SAT cell size and its related metabolic changes.

In response to an ongoing energy supply, adipose tissue is known to expand, due to enlargement of adipocytes [22]. Previous hypotheses suggested that low T levels in obese men may result from an up-regulated aromatase activity in the expanded adipose tissue, followed by elevated E_2_ levels which suppress the HPG axis [3, 4]. Studies however, which examined the role of aromatase and/or elevated E_2_ levels in obesity, reported conflicting results [23–25]. In males, this is one of the few studies evaluating aromatase expression in adipose tissue. Differences in SAT aromatase expression could not be established between obese men (with or without type 2 diabetes) and normal-weight men. Furthermore, we could not determine significant results concerning visceral adipose tissue (VAT) aromatase expression (data not shown), though SAT aromatase expression only was used for remaining analysis considering the generally known higher expression of aromatase in subcutaneous versus visceral adipocytes [26]. It has previously been suggested that SAT aromatase expression was associated with generalized obesity as described by BMI, though Wake et al. [24] could not find any association between SAT aromatase and abdominal obesity with which obese men are mostly characterized with. Some studies have reported data that counteract with the increased aromatase activity hypothesis. Firstly, Dhindsa et al. [7] reported lower E_2_ levels, measured with LC–MS/MS, excluding down-regulation by elevated E_2_ of the HPG axis as the main cause for low T levels in men with type 2 diabetes. Similar findings have been reported by Tajar et al. [27] who used LC–MS/MS to determine E_2_ as well. Finally, obese males have been described to have lower SHBG levels, which also counteracts with the increased aromatase activity hypothesis resulting in elevated E_2_ levels since E_2_ is known to normally stimulate SHBG production [28].

In the present study, we found, apart from an inverse correlation between (free) T and SAT cell size in univariate analyses, an inverse association of (free) T levels with TG and insulin sensitivity (HOMA-IR). Inverse associations between T levels and TG are consistent with the findings of previous studies [29, 30]. Elevated circulating TG levels have been suggested to be an indicator of metabolic derangement, associated with both glucose intolerance and increased amounts of VAT [31–33]. The inverse link between sex steroids and lipids has been described previously, suggesting a protective role of T and E_2_ for the cardiovascular system [34]. Pitteloud et al. reported an inverse association between human chorionic gonadotropin (HCG)-induced T secretion by Leydig cells and insulin sensitivity (measured by hyperinsulinemic euglycemic clamp) among men with various degrees of glucose tolerance. Thus, Leydig cell function is suggested to be altered in insulin-resistant men, resulting in decreased T secretion [35]. Another study reported a diminished T response to HCG in obese men, which correlated with baseline leptin levels [36]. Furthermore, Sertoli cell function was also suggested to be impaired in obese insulin-resistant men, since two Sertoli cell markers, inhibin B and anti-Müllerian hormone, were found to be lower in obese versus control men [37]. The inverse correlation between (free) T, adipocyte cell size, and HOMA-IR as well as circulating TG in this study also supports the findings of Pitteloud et al. [35]. On multivariate regression analysis, however, SAT cell size remained the only metabolic parameter that was independently related to (free) T. Adipocyte cell size has been recognized for many years as an important parameter in the pathogenesis of metabolic derangement. Enlarged subcutaneous adipocytes have been shown to predict type 2 diabetes in a prospective cohort, independent from clamp-measured insulin sensitivity [38, 39]. It has been shown that insulin-sensitive though severely obese individuals had a smaller adipocyte size compared to an equally obese but insulin-resistant group matched for age, sex, and body fat, suggesting that mechanisms beyond obesity *per se* determine the pathological metabolic consequences in obesity [40]. Furthermore, adipocyte cell size has been associated with low-grade systemic inflammation and macrophage accumulation in adipose tissue [22, 41]. Our data emphasize the importance of enlarged SAT cell size and related metabolic changes as a potential determinant of low T levels in obese men. However, these findings derive from a cross-sectional study, limiting conclusive statements on causality.

Our results suggest a direct negative impact of adipocytes, but not IR, on free and total T levels in obese men. The influence of adipokines, which are regulatory products of adipose tissue, on reproductive hormones and function has been repeatedly reported [42–44]. For instance, high adiponectin levels (typical of normal weight) have recently been found to regulate Leydig cell steroidogenesis and T secretion, through the transcriptional regulation of steroidogenic genes [44]. Expanded adipose tissue is known to differentially secrete its adipokines, leading to the release of more pro-inflammatory and less anti-inflammatory adipokines in case of obesity. A potential explanation of low (free) T levels could thus be a pro-inflammatory status in testes secondary to a deregulated adipokine secretion pattern, as enlarged adipocytes have been related to a systemic inflammatory state [41, 45]. Some inflammatory mediators, such as the adipokines TNF-α and leptin, have been shown to negatively influence HCG-induced T secretion directly from Leydig cells as well as to disturb Sertoli cell function [35, 42, 43]. Especially, leptin has been indicated as an important mediator in the development of reduced androgens in male obesity, as both influences on the HPG axis and direct testicular effects were reported by in vitro and in vivo studies [36, 46–48]. A recent study reported the suppression of HCG-induced T secretion in primary Leydig cells after the addition of chemerin, a novel adipokine of which elevated levels have been associated with both obesity and diabetes in humans [49]. In addition, inflammatory mediators have also been shown to suppress the HPG axis [50, 51]. The latter could explain the assumed disturbances in HPG axis, since primary testes injury normally is accompanied by an up-regulated gonadotropin release which cannot be demonstrated in our study cohort. Vermeulen et al. as well as Giagulli et al. have indicated these alterations in feedback regulation of gonadotropins by determining LH pulse amplitudes in obese men [4, 52].

Finally, bariatric surgery normalized BMI, TG, HOMA-IR, T, SHBG, and FSH values in the prospective part of our study. Our findings are consistent with previously reported data, which found improved BMI and T levels in men after bariatric surgery together with a restored sexual function and fertility. In accordance with previous studies, we found no changes in LH levels before and after surgery. Despite the use of the dialysis method, we found no changes in free T levels after GBS, in contrast to studies that used less precise methods such as calculated free T from total T [15, 53, 54]. This may suggest that a change in SHBG is mainly responsible for the increase in T, instead of recovery of the initial causative factors. Another explanation is the limited number of patients willing to participate in the follow-up part of the study, which could affect the detection of small changes. Longitudinal studies on a larger scale are needed to confirm these results. Recently, when bariatric surgery in hypogonadal subjects was compared to eugonadal men however, increase in (free) T levels after surgery had only been observed in hypogonadal men in combination with a more pronounced reduction in waist circumference (a marker of abdominal adiposity of these men). Furthermore, this study showed lower E_2_ levels at baseline in hypogonadal versus eugonadal obese men, also minimizing its role as a determinant of T levels in obese men [54].

The present study has the benefit of LC–MS/MS methods to determine T levels compared to previous commercial radioimmunoassay kits, leading to more reliable results, though larger studies are needed to confirm the findings. Another limitation of this study is the lack of information on adipose tissue aromatase activity in addition to the expression analysis, because SAT samples were frozen or fixated.

In conclusion, low T levels in obese men inversely associate with SAT cell size, HOMA-IR, and TG levels and not with adipose tissue aromatase expression, suggesting obesity-related metabolic disturbances to be more important for explaining the T levels in obese men. Further research should be directed at primary T secretion failure of the testis.
